# Exploring the feasibility and impact of positive psychology-motivational interviewing interventions to promote positive affect and physical activity in type 2 diabetes: design and methods from the BEHOLD-8 and BEHOLD-16 clinical trials

**DOI:** 10.1080/21642850.2020.1815538

**Published:** 2020-09-14

**Authors:** Juliana Zambrano, Christopher M. Celano, Wei-Jean Chung, Christina N. Massey, Emily H. Feig, Rachel A. Millstein, Brian C. Healy, Deborah J. Wexler, Elyse R. Park, Julia Golden, Jeff C. Huffman

**Affiliations:** a Department of Psychiatry, Massachusetts General Hospital, Boston, MA, USA; b Harvard Medical School, Boston, MA, USA; c Department of Neurology, Massachusetts General Hospital, Boston, MA, USA; d Department of Medicine (Endocrinology), Massachusetts General Hospital, Boston, MA, USA

**Keywords:** Motivational interviewing, physical activity, positive affect, positive psychology, type 2 diabetes

## Abstract

**Background:** Physical activity among those with type 2 diabetes (T2D) is independently associated with superior medical outcomes, but existing behavioral interventions have not led to widespread increases in activity in this population. A remotely delivered intervention that targets well-being constructs associated with greater activity and assists in the creation of specific physical activity goals has the potential to improve activity and outcomes in T2D.

**Objective:** To outline the rationale and methods of two studies designed to assess the impact and optimal duration of a combined positive psychology-motivational interviewing (PP-MI) intervention for inactive persons with T2D.

**Methods:** We conducted trials studying 8-week (BEHOLD-8;) and 16-week (BEHOLD-16;) phone-delivered interventions, compared to attention-matched control conditions. In a two-step randomization design, participants were allocated randomly first to study (BEHOLD-8 or BEHOLD-16), then to study condition within study. The primary aims in both trials were feasibility (rates of session completion) and acceptability (participant session ratings), with additional aims examining intervention effects on accelerometer-measured physical activity, psychological measures, and health-related metrics (e.g. vital signs). Main analyses, currently being conducted, will utilize mixed effects models between study conditions, and secondary analyses will utilize the same models to compare the 8- and 16-week PP-MI interventions on feasibility and impact.

**Results:** Enrollment and data collection have been completed for both trials (BEHOLD-8: *N* = 60; BEHOLD-16: *N* = 70), and data analysis is ongoing to assess feasibility and acceptability within study, as well as the relative feasibility and acceptability of the PP-MI interventions across the two studies. We will also explore impact on clinical outcomes between groups.

**Conclusions:** This design will address how intervention content (i.e. PP elements vs. no PP elements) and intervention duration (8 weeks vs. 16 weeks) affect feasibility, acceptability, and impact, allowing intervention optimization before a next-step larger clinical trial.

**Trial registration:** ClinicalTrials.gov identifier: NCT03150199; NCT03001999.

## Introduction

Type 2 diabetes (T2D) affects 29 million Americans (Benjamin et al., 2019), is the 7th leading cause of death in the U.S. (Matthews, Kirk, Macmillan, & Mutrie, 2014), and is a major risk factor for cardiovascular disease (Huo et al., 2016). Among patients with T2D, physical activity is associated with better glycemic control, reduced rates of T2D complications, and lower rates of all-cause mortality, independent of other health behaviors (Colberg 2016). Despite these benefits, the majority of T2D patients fail to meet recommended guidelines for physical activity (Morrato, Hill, Wyatt, Ghushchyan, & Sullivan, 2007). Intensive multicomponent programs for physical activity and other health behaviors in T2D have led to some benefits in research trials (Avery, Flynn, van Wersch, Sniehotta, & Trenell, 2012; Goldstein, Whitlock, & DePue, 2004) but are difficult to implement in clinical settings due to their intensity and complexity (Carroll et al., 2015; Parikh et al., 2010; Whittemore et al., 2009). Hence, there is a need to develop programs that are simple, scalable, and powerful enough to meaningfully promote health behaviors in persons with T2D.

Telephone-delivered motivational interviewing (MI) is a promising alternative to in-person programs. MI is an evidence-based, patient-centered counseling strategy designed to enhance intrinsic motivation for change (Miller & Rollnick, 2012b). It can be delivered remotely with good fidelity (Ingersoll et al., 2015; Kealey et al., 2009) and is effective in changing health behaviors (Christie & Channon, 2014). However, MI alone may not be enough. The effects of MI alone on physical activity in T2D have been relatively small (Soderlund, 2018), and engagement with MI and related interventions appears to be particularly diminished among individuals with low expectations of improvement or low overall optimism (Avery et al., 2012).

Interventions that promote psychological well-being also have the potential to increase physical activity among those with T2D, as well-being-related constructs (e.g. optimism, positive affect) are prospectively and independently linked with greater physical activity in T2D and other populations, independent of sociodemographics, medical factors, and the adverse effects of depression (DuBois et al., 2012; Giltay, Geleijnse, Zitman, Buijsse, & Kromhout, 2007; Huffman et al., 2016; Scheier et al., 1989; Steptoe, Wright, Kunz-Ebrecht, & Iliffe, 2006). Positive psychology (PP) interventions, which focus on the deliberate and systematic promotion of positive cognitive and emotional states through easy-to-complete exercises (e.g. expressing gratitude, using personal strengths) (Bolier et al., 2013), are particularly promising, as they can be delivered remotely, require minimal provider training (Bolier et al., 2013; Huffman et al., 2011; Schueller & Parks, 2012), and have led to improved well-being and increases in physical activity and other health behaviors among patients with medical illness (Ogedegbe et al., 2012; Peterson et al., 2012). PP could therefore be an effective and easy-to-deliver component of an intervention to increase physical activity in T2D patients.

**Figure 1.**
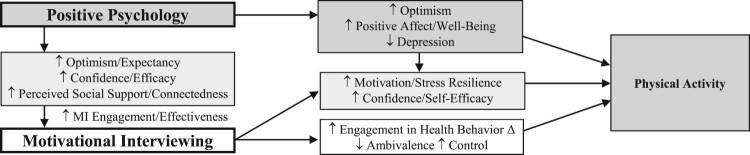
Conceptual model outlining potential mechanisms by which PP-MI may increase physical activity.

Given the potential benefits of both PP and MI, a combined PP-MI intervention could promote physical activity through the independent and combined effects of its two components (see model, Figure 1). Drawing from PP-based intervention projects in patients with coronary artery disease and other illnesses, and utilizing a multistep process for intervention development using the NIH Stage Model (Onken, Carroll, Shoham, Cuthbert, & Riddle, 2014), we developed a telephone-delivered, PP-MI program to promote well-being and physical activity in patients with T2D in the Boosting Emotions and Happiness in Outpatients living with Diabetes (BEHOLD) project. In a single-arm proof-of-concept trial (*N* = 10), this intervention was feasible, acceptable, and associated with pre–post improvements in well-being constructs and physical activity (Celano et al., 2019).

As a next step, we aim to optimize the PP-MI program by examining the feasibility, acceptability, and preliminary efficacy of its components and by determining its optimal duration. Accordingly, we are conducting two concurrent randomized pilot trials of the PP-MI intervention. In the first (BEHOLD-8), we are testing an 8-week version of the telephone-delivered PP-MI intervention, compared to an attention-matched MI-enhanced behavioral counseling condition. We chose this control condition to assess the incremental benefits of the PP content given that both groups would obtain MI related content and skills. In the second, (BEHOLD-16), we are comparing a 16-week PP-MI intervention to an attention-matched health education condition. We chose this control condition to allow cross-study assessment that would explore the relative feasibility and benefits of PP-MI, MI, and health education. Participants are first randomly allocated to either the 8-week (BEHOLD-8) or 16-week (BEHOLD-16) study, and then are allocated to the PP-MI or attention-matched control condition within that study. To match for attention, all conditions had phone sessions at the same intervals and duration, utilized written treatment manuals specific to the study condition, provided weekly activities for participants to complete on their own, and included the provision of pedometers to promote physical activity.

This pair of studies will allow us to examine the effects of intervention content (e.g. does the use of PP elements, versus no PP elements, lead to a more feasible and well-accepted program?) within the studies, and it will allow us to explore differences in feasibility and impact related to PP-MI intervention duration (e.g. does an 8 week or 16 week program appear to be more feasible, better-accepted, or potentially more effective?) We have completed enrollment and follow-up assessments for these trials; results are pending. In this article, we will outline the rationale, specific aims, detailed methods, and analysis plan for these two trials.

## Methods

BEHOLD-8 and BEHOLD-16 are randomized controlled pilot trials designed to examine the feasibility, acceptability, and preliminary efficacy of 8 and 16-week versions of a PP-MI intervention, compared to attention-matched, clinically relevant control conditions. All participants provided written informed consent prior to enrollment, with consent addressing randomization to study/intervention length and allocation to different intervention content within each study. Institutional Review Board approval was obtained prior to initiation of the studies (IRB approval #s: 2017P-001597 and 2017P-001113), and both studies were pre-registered on ClinicalTrials.gov (NCT03150199/NCT03001999). The study was conducted at Massachusetts General Hospital, an urban academic medical center in Boston, Massachusetts, USA.

In BEHOLD-8, an 8-week phone-delivered PP-MI intervention is compared to a time-matched MI-alone program in 60 inactive adults with T2D. In BEHOLD-16, the PP-MI intervention is delivered over 16 weeks, this time compared to a time- and attention-matched health-education intervention in 70 inactive adults with T2D. Study outcomes are measured at baseline, in a follow-up visit at the end of intervention (8 and 16 weeks), and in a follow-up phone call 8 weeks after ending the intervention (16 and 24 weeks, respectively).

We selected 8 and 16-week PP-MI interventions to attempt to find a balance between feasibility and longer-term impact. Prior PP-based interventions by our team and others in medical settings have had durations between 5–16 weeks (Carrico et al., 2015; Cheung et al., 2017; Duque, Brown, Celano, Healy, & Huffman, 2019; Nikrahan et al., 2019), and in prior PP-MI interventions we found that approximately 12 weeks appeared to find an adequate balance between tolerability and impact (Huffman et al., 2019). Having interventions lasting 8 and 16 weeks (i.e. with an 8-week difference in intervention duration) in the two studies would allow us to explore the impact of intervention duration on feasibility, acceptability, and impact between the 8- and 16-week PP-MI interventions across the studies.

**Figure 2.**
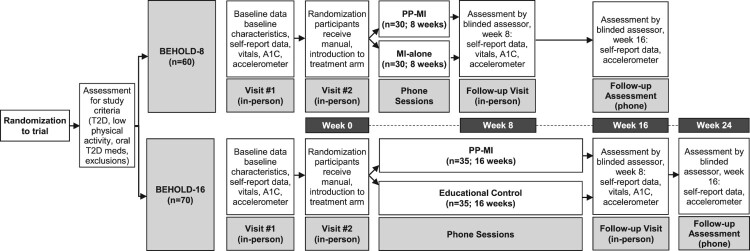
Assessment procedures.

These pilot trials have been concurrently conducted, using identical study criteria and recruitment from the same patient pool (see Figure 2). Allocation of participants was conducted first by study (to BEHOLD-8 and BEHOLD-16), and then to study condition, allowing us to compare effects both within and across studies. Main analyses will be performed within each trial (with feasibility and acceptability the primary outcomes examined in both trials). Secondary analyses will compare the 8- and 16-week PP-MI interventions across studies on feasibility, acceptability, and efficacy metrics.

### Trial design

The PP-MI intervention was developed based on a theoretical model that focuses on a multipronged approach to the promotion of physical activity. The intervention is composed of two components. The PP component focuses specifically on increasing psychological well-being through structured, validated PP activities and application of skills used in those activities to boost well-being in daily life. The MI component utilizes techniques from MI to resolve ambivalence, set goals, and reduced barriers to completing physical activity. In this model, it is postulated that a combined PP-MI intervention will promote physical activity through the independent and combined effects of its two components (see Figure 1). First, the two components should improve physical activity separately. The MI component should lead to greater intention to change, less ambivalence, and greater feelings of control (Bean et al., 2015; Smith, Kratt, & Mason, 1997), which are associated with increased physical activity (Boudreau & Godin, 2014; Moreau, Gagnon, & Boudreau, 2015; Plotnikoff, Trinh, Courneya, Karunamuni, & Sigal, 2011), while the PP component can directly promote positive affect and optimism and reduce depression (Bolier et al., 2013; Cohn, Pietrucha, Saslow, Hult, & Moskowitz, 2014; Meevissen, Peters, & Alberts, 2011), all of which are linked to more physical activity (Emmons & McCullough, 2003; Giltay et al., 2007; Peterson et al., 2012; Steptoe et al., 2006). Both MI (Bennett, Young, Nail, Winters-Stone, & Hanson, 2008; Gourlan, Sarrazin, & Trouilloud, 2013; Lilienthal, Pignol, Holm, & Vogeltanz-Holm, 2014; Martins & McNeil, 2009) and the effects of PP on mood, positive affect, and optimism (DiMatteo & Haskard-Zolnierek, 2011; Fredrickson, 2001; He, Cao, Feng, Guan, & Peng, 2013; Majer, Jason, & Olson, 2004; Scheier et al., 2007) are likely to improve self-efficacy, confidence, resilience, and motivation, which in turn could translate to increased activity (Dutton et al., 2009; Littlecott, Moore, Moore, & Murphy, 2014; Plotnikoff et al., 2011; Sweet et al., 2009).

Finally, the PP element of the intervention can also increase engagement and impact related to the MI intervention. The PP intervention component can lead to increases in optimism and outcome expectancy (King, 2001; Meevissen et al., 2011), confidence and self-efficacy (Lee, Robin Cohen, Edgar, Laizner, & Gagnon, 2006; Smeets, Neff, Alberts, & Peters, 2014), and perceived social support and interpersonal connectedness (Fredrickson, Cohn, Coffey, Pek, & Finkel, 2008; Lyubomirsky & Layous, 2013). These individual- and social-level constructs, in turn, are associated with greater engagement in, and efficacy of, MI and other health behavior interventions (Goossens, Vlaeyen, Hidding, Kole-Snijders, & Evers, 2005; Joseph et al., 2010; Scheier et al., 2007; Schmiege, Feldstein Ewing, Hendershot, & Bryan, 2011), and therefore PP activities may boost the effects of MI on physical activity. These complementary effects of PP and MI should be much more powerful in this vulnerable population than either approach alone. Prior work combining psychological and behavioral interventions has been successful in medical populations (Safren et al., 2014), and MI has been integrated well with other treatments (Ismail et al., 2010; Smith et al., 1997). Such work has specifically included PP-MI interventions in medical populations (Celano et al., 2019; Celano, Albanese et al., 2018; Huffman et al., 2019).

The PP-MI intervention has been created and tested using elements of the NIH Stage Model (Onken et al., 2014) and the Multiphase Optimization Strategy (MOST) treatment development process, a method for preparing, evaluating, and optimizing multicomponent behavioral interventions (Collins, Murphy, Nair, & Strecher, 2005). In the initial phase, the intervention was developed and customized for T2D patients with low baseline physical activity using an existing PP-MI program in patients with cardiovascular disease (Huffman et al., 2017), qualitative interviews with T2D patients (Madva et al., 2018), and input from experts in T2D. Next, the feasibility and acceptability of the intervention was assessed in a single-arm proof-of-concept trial (*N* = 10). Session completion rates were high, participants rated the PP-MI content and sessions as useful (mean rating = 9.1/10, SD = 1.5), and PP-MI was associated with improved adherence to physical activity and overall self-care (Celano et al., 2019).

A next-step goal, prior to testing the PP-MI intervention in a large, well-powered efficacy trial at a future date, was to confirm the intervention’s feasibility and potential impact compared to a control condition, and also to determine the optimal duration of such an intervention to find the best balance across participant burden, cost-effectiveness, and impact, in the current trials. By performing a pair of randomized controlled trials, in which participants are randomized both to study and to intervention within the study, we are able to compare the 8- and 16-week PP-MI interventions both to their control conditions and to each other. Performing analyses both within and between studies will allow us to identify the intervention length and complexity associated with the greatest feasibility and improvement in MVPA and other key outcomes.

In both studies, we hypothesized that participants in the PP-MI conditions would reach *a priori* benchmarks for feasibility and acceptability. We also expected the PP-MI interventions to lead to small to medium effect size improvements in MVPA and other psychological and medical outcomes, compared to their control conditions, given the prior links between well-being and physical activity and our successful proof-of-concept trials (Celano et al., 2019; DuBois, Millstein, Celano, Wexler, & Huffman, 2016; Huffman et al., 2017). We were at equipoise about relative benefits of the two PP-MI conditions with regard to feasibility and impact.

### Participants

#### Inclusion criteria

Eligible participants were adults (age ≥ 18 years) treated in an MGH-affiliated outpatient clinic with:

1. Diagnosis of T2D (i.e. meeting American Diabetes Association [ADA] criteria (American Diabetes Association, 2013) for T2D [e.g. hemoglobin A1c ≥6.5% and/or fasting glucose ≥126 mg/dl]), confirmed by their diabetes clinician and/or medical record review.
2. Low physical activity, defined as ≤150 min/week of MVPA (corresponding to American Diabetes Association recommendations for moderate or greater intensity aerobic physical activity). Physical activity over the past week (or a typical week, if the past week was atypical) was measured using the MVPA-specific questions from the International Physical Activity Questionnaire (IPAQ), a validated tool used in medical cohorts (Booth et al., 2003; Lee, Macfarlane, Lam, & Stewart, 2011).

#### Exclusion criteria

Patients were excluded due to: (1) cognitive impairment precluding consent or meaningful participation, assessed using a six-item screen developed to assess cognitive capacity to participate in research studies (Callahan, Unverzagt, Hui, Perkins, & Hendrie, 2002), (2) lack of phone availability, (3) inability to read/write in English, (4) medical conditions (e.g. severe arthritis) that precluded physical activity, or (5) current participation in mind–body programs, lifestyle intervention programs (e.g. cardiac rehabilitation), or other clinical trials.

### Study conditions and program content

Please see Appendix for a completed TIDieR checklist (Hoffmann et al., 2014) regarding the description of all study interventions. The same team of interventionists delivered interventions in all arms of both studies to reduce interventionist-specific effects on study outcomes.

#### Initial visit for all conditions

During the initial in-person baseline study visit, participants completed baseline study outcome measures, including self-report assessments, vital signs, and venipuncture (for hemoglobin A1c). At this visit, participants received hip-worn Actigraph GT3X+ accelerometers to wear during waking hours for 1 week to measure baseline physical activity and then returned for a second in-person baseline study visit. During this second visit, adequate accelerometer wear time was confirmed, participants were assigned to a treatment condition, and study interventionists introduced participants to their appropriate conditions (Figure 2). All participants received a study manual corresponding to their treatment condition (intervention manuals available from authors) and an Omron HJ-520 pedometer to track physical activity.

#### BEHOLD-8 PP-MI intervention

Following receipt of the treatment manual and completion of the in-person session at the second in-person baseline study visit, the remainder of the intervention was completed by phone. Each week, the PP portion of phone sessions (15 mins) was structured to include: (i) review of the prior week’s assigned PP exercise with the participant to explore its impact on well-being, (ii) discussion about how to translate the PP exercise skills from that activity into daily life (e.g. related to physical activity or other goals), and (iii) assignment of the next exercise via co-review of the treatment manual, with a discussion of the rationale and details of the exercise. Participants then completed the PP exercise independently during the week and wrote about the exercise and its effects. The MI portion of calls (15 mins) assessed participant motivation to increase physical activity and then planned action steps or cognitive work to boost motivation, using the structure and principles of MI.

PP and MI components were delivered separately within sessions (rather than intertwined) based on our experience, participant feedback, and pilot work (Celano et al., 2019; DuBois et al., 2016; Huffman et al., 2017). For example, participants did not need to write their gratitude letter to a person involved in their diabetes care; instead, they could focus on well-being in any life domain, to more broadly promote motivation, optimism, and positive affect.

The PP exercises (see Table 1) were selected based on their performance in prior studies and were adapted to T2D population in terms of the rationale for completing the exercises and their potential impact on T2D (Celano et al., 2019; Huffman et al., 2017). Between phone sessions, participants completed and wrote about the assigned PP exercise, as well as the positive thoughts and emotions they experienced while completing the exercise. Intervention consistency was maintained by keeping exercises assigned in the same sequence for all participants.

**Table 1.** BEHOLD-8 PP-MI intervention components (9 sessions over 8 weeks).

Session	PP component**	MI component***
1*	*Gratitude for positive events* Participants identify three positive events that have occurred in the past week and reflect on their feelings as they recall and describe these events.	*Moving for better health/activity tracking* Participants review their current activity level, set an overall physical activity goal, assess the importance and confidence of reaching this goal, and consider the pros/cons of increasing activity.
2	*Gratitude letter* Participants write a letter of gratitude thanking a person for their support or kindness.	*Setting a SMART physical activity goal* Participants learn about and set a SMART (specific, measurable, attainable, relevant, and time-based) physical activity goal.
3	*Enjoyable and meaningful activities* Participants complete three activities: an enjoyable activity alone, an enjoyable activity with another person, and a meaningful activity alone or with others.	*Barriers and problem solving* Participants consider past and anticipated barriers and facilitators to physical activity. They continue to refine/set new goals.
4	*Gratitude and Meaning skills application* Participants select a useful activity from the prior three weeks, consider how to adapt the activity to daily life, and develop a plan to utilize it regularly.	*Using social resources* Participants identify social resources for physical activity.
5	*Recalling past success* Participants recall an event in which they experienced success, then write about the event, their contribution to the success, and positive feelings elicited by recalling it.	*Finding new routes* Participants complete a neighborhood walk audit and identify new walking routes.
6	*Using personal strengths* Participants undergo a brief assessment of personal strengths, then find a specific new way to use one of their ‘signature strengths’ in the next 7 days.	*Using neighborhood resources* Participants identify neighborhood resources for physical activity.
7	*Using personal strengths (part 2)* Participants repeat the personal strengths assessment, select a second strength, and find a specific new way to use it in the next 7 days.	*Managing ‘slips’* Participants learn tips about the management of ‘slips’ (i.e. temporary reductions) in physical activity.
8	*Acts of kindness* Participants use the strength of altruism by planning and then completing three acts of kindness toward others, all performed within a single day.	*Reducing sedentary time/taking breaks* Participants identify daily sedentary time and discuss strategies for reducing sedentary time, including regular standing breaks.
9	*Skills application + future planning* Participants select an activity from the prior four weeks and develop a plan to utilize this skill—and additional skills from the program – this week and beyond.	*Reviewing progress/considering the future* Participants review their accomplishments and create a plan for physical activity for the near future.

* In-person study visit.

** Each week, in the PP component of the intervention, the study interventionist assists the participant in considering how to apply skills from the PP exercise in daily life.

*** Each week in the MI component of the intervention, the interventionists also use the 5A’s model to: (a) ask participants about their physical activity goals, (b) advise them about current activity guidelines and/or refer them to their treatment team, (c) assess readiness to set an activity goal, (d) assist participants in clarifying their goals and problem-solving barriers to reaching those goals, and (e) arrange for the next session by summarizing the participant’s plan for the physical activity goal and scheduling the next session.

After review of the PP exercises during the phone session, the interventionist applied MI techniques to specifically target physical activity goal setting. The MI portion of sessions utilized the ‘5As’ strategy (Ask, Advise, Assess, Assist, Arrange (Miller & Rollnick, 2012a)) to evaluate and drive motivation to increase physical activity. The setting of explicit, proximal, and attainable goals with specific feedback was used to reinforce progress and enhance self-efficacy, based on social cognitive theory and the transtheoretical model of behavior change (Bandura, 2004; Bandura & Locke, 2003). Each phone session consisted of reviewing participants’ progress on their physical activity goal from the prior week, discussing weekly topics related to goal-setting and physical activities, and setting new physical activity goals for the next week. Interventionists and participants set goals that gradually increased in duration and/or intensity, with input from participants’ physicians when relevant to ensure safety. Weekly topics (e.g. activity tracking, managing slips) were designed to promote engagement with goal-setting and physical activity. As noted, participants were given a digital pedometer (Omron HJ-520) to promote and track physical activity.

#### BEHOLD-8 MI-enhanced behavioral counseling control condition

Participants randomized to this attention-matched condition had weekly activities focused on T2D health education combined with MI-specific content divided into four major topic areas: diabetes self-care, medication adherence, physical activity, and diet (Table 2). An initial session focused on overall self-care, and subsequent modules focused on adherence to medication (2 sessions), physical activity (3 sessions), diet (2 sessions), and an overall review. During each phone call, interventionists used MI principles and related behavioral change theory approaches as in the MI portion of the PP-MI intervention (e.g. 5As strategy, setting of attainable goals) to enhance engagement in health behaviors, reviewed health education topics introduced in the previous week, identified ways to improve adherence, and introduced specific new weekly topics. Participants then worked toward health behavior goals, wrote about their progress in their manual, and reviewed this information at weekly calls, in a parallel manner to the PP-MI program. Because the MI-alone condition did not contain PP content, the full duration of the call was used to discuss weekly topics, goals, and barriers/facilitators to adherence in an expanded manner.

**Table 2.** BEHOLD-8 MI-based health behavior education Intervention components.

Session	Health Behavior
Part One: Diabetes Self-care	
1*	*How to Take Care of Your Diabetes* Participants learn about the importance of adherence to self-care behaviors (e.g. glucose monitoring, foot checks, eye exams) to reduce the risk of T2D complications.
Part Two: Medication Adherence	
2	*Taking Medications Regularly* Participants review the importance of medication adherence and learn about medications typically prescribed for T2D. Participants are encouraged to create a list of their current medications, as well as any questions they have for their physicians about their medications.
3	*Barriers to and Resources for Taking Medications Regularly* Participants identify barriers to medication adherence, problem-solve those barriers, and identify resources to help them maintain adherence to medications.
Part Three: Staying Active	
4	*The Importance of Staying Active* Participants review the cardiovascular benefits of physical activity, questions to ask their physician prior to starting physical activity, and ways to set goals related to physical activity.
5	*How You Can Stay Active* Participants identify one way in which they wish to change their physical activity. They discuss the importance of making the change and their confidence about being able to do so. Finally, they create a list of pros and cons for changing this behavior.
6	*Barriers to and Resources for Activity* Participants identify barriers to physical activity in their life and brainstorm ways to problem-solve around those barriers. Furthermore, they identify social, community-based, and equipment resources available to help them with physical activity.
Part Four: Having a Healthy Diet	
7	*Making Dietary Changes* Participants learn about the importance of reducing sugar, fat, and cholesterol in their diet. Next, they identify one way in which they wish to change their diet. They discuss the importance of making the change and their confidence about being able to do so. Finally, they create a list of pros and cons for behavior change.
8	*Barriers to and Resources for a Healthy Diet* Participants review how to properly read a food label and learn techniques to make healthy decisions while food shopping. They also identify barriers to a healthy diet, problem-solve those barriers, and identify resources that can help to improve their diet.
9	*Planning for the Future* Participants review the information learned over the course of the program and think of ways to remain healthy after the end of the program.

* during in-person session.

#### BEHOLD-16 PP-MI intervention

Similar to BEHOLD-8, participants received a treatment manual with weekly activities. Interventionists introduced the manual and treatment program during an in-person session covering the initial PP and MI exercises. Subsequent sessions were completed via phone over 16 weeks. There were 14 planned PP-MI sessions (Table 3) delivered over 16 weeks (to account for common scheduling issues given the length of the intervention based on our experience). Additional ‘bonus’ sessions were conducted if participants completed all 14 sessions prior to the end of week 16.

**Table 3.** BEHOLD-16 PP-MI intervention components (14 sessions over 16 weeks).

Session	PP component**	MI component***
1*	*Gratitude for positive events* Participants identify three positive events that have occurred in the past week and reflect on their feelings as they recall and describe these events.	*Moving for better health/activity tracking* Participants review their current activity level, set an overall physical activity goal, assess the importance and confidence of reaching this goal, and consider the pros/cons of increasing activity.
2	*Gratitude letter* Participants write a letter of gratitude thanking a person for their support or kindness.	*Setting a SMART physical activity goal* Participants learn about and set a SMART (specific, measurable, attainable, relevant, and time-based) physical activity goal.
3	*Capitalizing on Positive Events* Participants identify a positive event as it occurs in their daily life and then capitalize on it by telling another person, writing about it, or celebrating the event in some other way.	*Barriers and problem solving* Participants consider past and anticipated barriers and facilitators to physical activity. They continue to refine/set new goals.
4	*Gratitude skills application* Participants select a useful activity from the prior weeks, consider how to adapt the activity to daily life, and develop a plan to utilize it regularly.	*Reviewing and reflecting on physical activity* Participants review progress towards their overall activity goal, then refine their overall goal for the remainder of the program.
5	*Recalling past success* Participants recall an event in which they experienced success, then write about the event, their contribution to the success, and positive feelings elicited by recalling it.	*Finding new routes* Participants complete a neighborhood walk audit and identify new walking routes.
6	*Using personal strengths* Participants undergo a brief assessment of personal strengths, then find a specific new way to use one of their ‘signature strengths’ in the next 7 days.	*Using neighborhood resources* Participants consider neighborhood resources for physical activity.
7	*Using personal strengths (part 2)* Participants repeat the personal strengths assessment, select a second strength, and find a specific new way to use it in the next 7 days.	*Using equipment resources* Participants identify equipment-related resources that could promote activity.
8	*Using perseverance* Participants use perseverance to complete a specific goal that week.	*Using social resources* Participants identify social resources for physical activity.
9	*Using strengths in daily life* Participants select a useful activity from the prior three weeks, consider how to adapt the activity to daily life, and develop a plan to utilize it regularly.	*Reviewing and reflecting on physical activity* Participants review progress towards their overall activity goal, then refine their overall goal for the remainder of the program.
10	*Enjoyable and meaningful activities* Participants complete three activities: an enjoyable activity alone, an enjoyable activity with another person, and a meaningful activity alone or with others.	*Managing Slips* Participants learn tips about the management of ‘slips’ (i.e. temporary reductions) in physical activity.
11	*Acts of kindness* Participants use the strength of altruism by planning and then completing three acts of kindness toward others, all performed within a single day.	*Reducing sedentary time* Participants assess the amount of time they spend sitting during the day and discuss strategies for reducing sedentary time.
12	*The ‘Good Life’* Participants write about what their ‘good life’ would look like in the next year in one or more life domains.	*Standing breaks* Participants learn about standing breaks as a strategy to reduce sedentary time and set a standing break goal for the week.
13	*Focusing on Meaning in Life* Participants select a useful activity from the prior three weeks, consider how to adapt the activity to daily life, and develop a plan to utilize it regularly.	*Increasing your strength through exercise* Participants learn about strength training and strength-based physical activity goal.
14	*Skills application + future planning* Participants review their favorite exercises and make a plan for continuing to use their PP-based skills in the future.	*Reviewing progress/considering the future* Participants review their accomplishments and create a plan for physical activity for the near future.

* In-person study visit.

** Each week, in the PP component of the intervention, the study interventionist assists the participant in considering how to apply skills from the PP exercise in daily life.

*** Each week in the MI component of the intervention, the interventionists also use the 5A’s model to: (a) ask participants about their physical activity goals, (b) advise them about current activity guidelines and/or refer them to their treatment team, (c) assess readiness to set an activity goal, (d) assist participants in clarifying their goals and problem-solving barriers to reaching those goals, and (e) arrange for the next session by summarizing the participant’s plan for the physical activity goal and scheduling the next session.

The PP sessions had the same structure as in BEHOLD-8. In this program, PP exercises were grouped into three categories: gratitude-based, strength-based, and meaning-based activities, and at the end of each session, an integration session was included to further enhance participants’ ability to integrate PP practices into their daily life. A final session focused on planning for the future was also included.

The MI sessions in the BEHOLD-16 PP-MI intervention also had the same structure as BEHOLD-8. Additionally, ‘review and reflect’ sessions (at sessions 4, 9, and 14) corresponded with each integration session with the PP exercises and were used to further enhance motivation and commitment to physical activity throughout the course of the intervention. Participants also received pedometers and were mailed a graph of their weekly step counts following each review and reflect session.

#### BEHOLD-16 diabetes health education condition

Participants randomized to this attention- and time-matched condition completed weekly readings in a treatment manual and answered questions related to T2D health, self-care, and health behavior adherence (See Table 4). This condition had a parallel structure to the experimental arm to account for time-matching, with a treatment manual, weekly exercises, and weekly calls to review exercises. We chose to utilize an active attention-matched education condition (rather than a wait-list control) to increase the rigor of the trial (i.e. to explore whether the 16-week PP-MI intervention may have greater effects on psychological and behavioral outcomes than a fully-attention-matched condition focused specifically on diabetes), and to allow for cross-study analyses examining the relative benefits of PP-MI, MI, and health education. As in the BEHOLD-8 control condition, the intervention was divided into four sections, focusing on different important diabetes health-related topics: diabetes self-care, medication adherence, diet, and physical activity (Table 4). This control condition differed from the BEHOLD-8 control condition in that the BEHOLD-8 control condition utilized MI and related tools in parallel with the PP-MI intervention, while the BEHOLD-16 control condition provided education without utilizing MI. The sessions were composed of modules on overall self-care (4 sessions), medication adherence (3 sessions), diet (3 sessions), physical activity (3 sessions), and an overall review session.

**Table 4.** BEHOLD-16 Diabetes Education intervention component.

Session	Health Behavior
Part One: Self-monitoring and Healthy Behaviors	
1*	*Diabetes and Risk Factors* Participants learn about diabetes complications (e.g. neuropathy, retinopathy, nephropathy) and their modifiable and non-modifiable risk factors.
2	*The Importance of Self-Monitoring* Participants learn about the importance of monitoring blood glucose, blood pressure, and cholesterol.
3	*Monitoring your Blood Sugar* Participants learn how to monitor blood glucose, including how to track and interpret their results.
4	*Foot Care and Eye Exams* Participants learn about the importance of regular appointments with podiatrists and ophthalmologists to minimize diabetes complications.
Part Two: Medication Adherence	
5	*The Importance of Medications in Diabetes* Participants review pharmacologic treatments for diabetes, including oral and injectable medications.
6	*Keeping Track of Medications* Participants develop a system for tracking adherence to currently prescribed medications.
7	*Barriers to and Resources for Medication Adherence* Participants problem-solve barriers to and identify resources for medication adherence.
Part Three: Healthy Eating	
8	*The Importance of Healthy Eating in Diabetes* Participants learn about the important components of a healthy diet and the benefits of a healthy diet in diabetes.
9	*A Healthy Eating Plan* Participants learn information about practical ways to initiate a healthy eating plan.
10	*Barriers to and Resources for Healthy Eating* Participants problem-solve barriers to and identify resources for maintaining a healthy diet.
Part Four: Physical activity	
11	*The Importance of Physical Activity in Diabetes* Participants learn about the health benefits of physical activity.
12	*How You Can Become More Physically Active* Participants identify practical ways to increase physical activity and consider a physical activity goal.
13	*Barriers to and Resources for Physical Activity* Participants problem-solve barriers to and identify resources for engaging in physical activity.
14	*Planning for the Future* Participants review the information that was covered over the course of the program and make plans for continuing to engage in healthy behaviors in the future.

*during in-person session.

### Training and fidelity

All team interventionists were psychiatrists or PhD level psychologists. Once the intervention was adapted and refined for T2D, they received additional training from study experts in PP and MI (CC, EP) using a structured training program that involved didactic sessions, role play, review of recorded sessions, and supervised interviews. The same group of interventionists delivered all arms of both studies and all interventionist phone sessions were recorded. During the study, weekly hour-long meetings were led by PP and MI supervisors (CC, RM) to discuss progress of all enrolled participants and to review audio recordings of sessions. Supervising study staff rated a random selection of sessions in a structured manner for fidelity, using scales for the PP and MI components, and provided ongoing feedback to interventionists during the supervisory sessions. Supervisors worked to avoid between-condition contamination (e.g. using PP principles for patients in an MI-alone condition) via training, weekly session review, and fidelity ratings.

The fidelity scales utilized were derived from existing MI fidelity assessments (Moyers, Rowell, Manuel, Ernst, & Houck, 2016; Robb, Burns, Docherty, & Haase, 2011), along with structured scales for the PP component and for the control conditions that had been created and utilized in our prior work (scales available from authors (Celano, Albanese et al., 2018; Huffman et al., 2015)). For PP-MI, the scale for each component was composed of 7 items (14 total), and fidelity was defined as meeting fidelity scale criteria for all 14 items. For the control conditions, the form contained 7 items (including an item regarding cross-condition contamination [in the diabetes education condition] and an item about the incorporation of MI principles [in the MI-only control condition]) and fidelity was defined as meeting the defined criteria for all 7 items. Intervention supervisors provided direct feedback at interventionist meetings and/or by email to interventionists if the interventionist did not score all points on fidelity assessments in any reviewed session.

## Study outcomes

### Outcome measures

Data collected for both studies, including outcome measures, were identical. Participants’ baseline demographic and medical characteristics were collected at the first in-person baseline study visit and supplemented using the electronic medical record. Psychological, behavioral, functional, and health-related measures (e.g. hemoglobin A1c, vital signs) were obtained at the initial study visit and repeated at a post-intervention visit (8 weeks [BEHOLD-8] and 16 weeks [BEHOLD-16]). A follow-up phone assessment occurred eight weeks later to obtain final self-report measures (16 weeks [BEHOLD-8] and 24 weeks [BEHOLD-16]). Finally, at both the post-intervention and follow-up assessments, participants were mailed accelerometers to wear during waking hours for one week, to measure MVPA; wear time was confirmed by study staff upon their return, and accelerometers were re-worn if participants had less than 4 days of wear for 8+ hours required for adequate wear, as per published guidelines (Choi, Ward, Schnelle, & Buchowski, 2012; Copeland & Esliger, 2009).

#### Feasibility and acceptability (primary study aim)

For both studies, feasibility and acceptability were the primary study outcomes and were measured on an ongoing basis during the intervention. For this primary study aim, we focused largely on three main areas that are examined in feasibility studies: implementation, satisfaction, and perceived utility/appropriateness, as outlined in prior guidelines for the conduct of feasibility studies (Bowen et al., 2009). For feasibility, interventionists recorded the proportion of possible PP-MI phone sessions that were successfully completed, with completion defined as the participant both completing a PP exercise and setting a physical activity goal. For acceptability, participant ratings of session ease and utility were used. Each week, participants separately rated the ease and utility on 0–10 Likert scales (0 = very difficult/totally unhelpful; 10 = very easy/very helpful) of the prior week’s PP exercise and MI topic, for four total ratings per session. These ratings were provided by participants to study interventionists at weekly calls. We chose these specific metrics for feasibility and acceptability to match the same metrics used in several of our team’s prior studies of feasibility and acceptability in medical and psychiatric populations (Celano, Freedman, Beale, Gomez-Bernal, & Huffman, 2018).

#### Physical activity

The main health behavior target for both clinical trials was MVPA, given its links to reduced rates of diabetes complications, the development of cardiovascular disease, and mortality (Gebel et al., 2015; Kubota et al., 2017; Saint-Maurice, Troiano, Matthews, & Kraus, 2018). Overall physical activity (steps/day) and sedentary time were also measured, given their prospective links to superior cardiovascular outcomes (Dohrn, Kwak, Oja, Sjostrom, & Hagstromer, 2018; Loprinzi, 2017). All physical activity outcomes were measured by a waist-worn Actigraph GT3X+ accelerometer (Actigraph, Pensacola, FL). This device was chosen because it has been extensively validated and used in a wide range of patient populations (including our prior PP-MI trials) (Celano et al., 2019; Huffman et al., 2017). The accelerometer was worn for 7 days at baseline and both follow-up points. We used MVPA cutoff of 1952 counts/minute on the accelerometer as it the most commonly used criterion in a mixed aged population (Gorman et al., 2014), steps, sedentary time (<150 counts per minute), and non-wear time cutoffs were set per the literature (Choi et al., 2012; Copeland & Esliger, 2009; Lynch et al., 2016; Prince, Blanchard, Grace, & Reid, 2016).

#### Self-report measures

An additional key aim of the trials was to assess the impact of the PP-MI interventions on psychological and behavioral outcomes. The main psychological outcome was positive affect, measured using the positive affect items of the Positive and Negative Affect Schedule (PANAS) (Watson, Clark, & Tellegen, 1988), given that it was a proximal target for the intervention, is sensitive to change, and has been linked to health outcomes (Moskowitz et al., 2012; Moskowitz, Epel, & Acree, 2008). Additional measures included the Life Orientation Test-Revised (LOT-R) for dispositional/trait optimism (Scheier, Carver, & Bridges, 1994), the Hospital Anxiety and Depression Scale (HADS) for depression and anxiety in the medically ill (Bjelland, Dahl, Haug, & Neckelmann, 2002), the Brief Resilience Scale (BRS) (Smith et al., 2008) for resilience, the PROMIS-physical function 20-item scale (Bartlett et al., 2015) for physical function, and the Self-Efficacy for Exercise scale (SEE) for physical activity self-efficacy (Dougherty, Glenny, Kudenchuk, Malinick, & Flo, 2010; Resnick & Jenkins, 2000; Schneider et al., 2011), consistent with the project’s theoretical model.

#### Medical outcome measures (exploratory)

We explored group differences in systolic and diastolic blood pressure, BMI, and hemoglobin A1c as exploratory outcome measures. These outcomes were collected by trained research nurses at the Translational and Clinical Research Center, utilizing standardized protocols, at baseline and the post-intervention follow-up visits.

### Randomization

Potential participants were identified by searching the hospital’s electronic health record data registry for patients with T2D who were followed in an affiliated outpatient clinic, and potential eligibility was confirmed with outpatient providers. Using a Zelen-type approach for initial allocation to study (Zelen, 1979), potentially eligible patients were randomly assigned to either the 8-week (BEHOLD-8) or 16-week (BEHOLD-16) study using a random number generator; they would later be randomized to experimental or control conditions within study post-consent (see below). Next, the patients received letters informing them about the study and allowing them to opt out of phone calls to provide them more information about their assigned study. Study staff then conducted phone calls with those who did not opt out to describe the study and its procedures, risks, and potential benefits, and to assess for eligibility. Interested and eligible patients were scheduled for an initial in-person visit at the hospital’s Translational and Clinical Research Center, and a written IRB-approved consent form was sent to them for review prior to the visit. During this initial in-person baseline study visit, participants provided informed consent.

### Treatment allocation

As noted, patients had been randomly assigned to study (BEHOLD-8 or BEHOLD-16) prior to initial contact. Following completion of informed consent, they were then assigned to treatment condition within their study. In BEHOLD-8 (*n* = 60), participants were assigned to a treatment condition using allocation by minimization (Scott, McPherson, Ramsay, & Campbell, 2002), a dynamic allocation technique to achieve balance of several key clinical variables (i.e. age, gender, medical comorbidity, and baseline MVPA) (Han, Enas, & McEntegart, 2009; Taves, 1974). This technique was utilized, as recommended by the grant review section, in BEHOLD-8 due to the somewhat small sample (which was also modestly smaller than BEHOLD-16) to avoid chance imbalance in groups that can occur in smaller trials. The first 16 participants were assigned to a study condition via simple randomization (using the same methods as BEHOLD-16 outlined below). For all subsequent enrollees, allocation of participants to study condition employed a dynamic automated minimization algorithm (Scott et al., 2002) to ensure balance for age dichotomized at 65, gender, MVPA (≤ 10 and >10 min of MVPA/day) and medical comorbidity (Charlson comorbidity index (Charlson, Pompei, Ales, & MacKenzie, 1987) dichotomized at ≤4.0 and >4.0) variables, using established methods (Saghaei, 2011; Scott et al., 2002; Taves, 1974). Cutoffs for activity and comorbidity were generated based on median scores for the initial set of participants randomized to the groups. Participants and study staff learned of the participant’s study condition concurrently.

Participants in BEHOLD-16 (*n* = 70) were randomly assigned to the PP-MI or diabetes health education condition. Prior to study initiation, a research coordinator not involved in the project used a random number generator to create a random sequence of treatment assignments. Slips of paper were put into opaque envelopes; these were opened in sequence by study staff following the completion of baseline measures and confirmation of adequate wear time, ensuring that participants and study staff learned of the study assignment contemporaneously.

## Statistical methods

Descriptive statistics (means, standard deviations, proportions) will be used to summarize baseline participant characteristics, and between-group differences in baseline characteristics will be calculated using chi-square analyses, Fisher’s exact tests, and independent samples t-tests.

### Within-study evaluations (e.g. PP-MI vs. MI-based health education in BEHOLD-8)

#### Feasibility and acceptability:

For our primary aim of feasibility and acceptability, we will assess the mean number of PP-MI phone sessions completed by participants; we will also calculate the proportion of participants who completed at least half of intervention sessions, as a marker of receiving adequate intervention content during the intervention period. The means and standard deviations (SD) of the 0–10 ease/utility scores of the PP and MI component of each session will be used to assess acceptability, for a total of 4 separate mean scores. We set a benchmark of 70% completion of assigned sessions and mean ratings of 7.0/10 on ease and utility *a priori* for feasibility and acceptability, based on similar interventions and scales (Celano, Albanese et al., 2018; Huffman et al., 2019).

#### Psychological, behavioral, and medical outcomes:

Between-group differences in change from baseline on physical activity metrics and other study outcome measures will be assessed using mixed effects regression models with an unstructured covariance matrix. We selected these models because they allow for the inclusion of all participants, including those with missing data (Blackwell, de Leon, & Miller, 2006). Both sets of analyses will also adjust for the allocation variables (age, gender, comorbidity, and baseline MVPA) used in the BEHOLD-8 study, as planned for the BEHOLD-8 study and to maintain consistency in analyses between the two studies.

Alpha for between-group comparisons was set at *p* = .05 to denote statistical significance for main outcomes and secondary outcome measures of MVPA, steps, and positive affect. Given that these initial studies were not designed to detect between-group differences in other outcomes, we will estimate between-group effect size differences (utilizing Cohen’s *d*, defined as change in the outcome measure compared to control group, divided by the standard deviation of the residual of the mixed effects model) of PP-MI for each outcome.

Additionally, to assess the possible effect of change in positive affect (measured by PANAS) as a mediator of change in MVPA, we will use a series of linear regression analyses to assess the total intervention effect on change in MVPA, the direct effect on change in MVPA, and the indirect effect on change in MVPA mediated through the PANAS. In addition to mediation analysis (Baron & Kenny, 1986), bootstrapping will be used to create a 95% confidence interval for these total, direct, and indirect effects. Secondarily, we will examine the potential mediating effects of exercise self-efficacy (measured by SEE) on MVPA, utilizing the same statistical approach.

##### Power considerations

Sample sizes were calculated for feasibility and acceptability based on rates of session completion in prior related interventions that had beneficial effect on health behaviors in clinical populations (Huffman et al., 2011; Huffman et al., 2015). We conducted the following power analyses *a priori* to confirm the adequacy of the samples used in these studies. Using a 84% completion rate by three-quarters of subjects in these prior studies, we had a minimum of 80% power (with 30 participants in each assigned condition for BEHOLD-8, and 35 per condition in BEHOLD-16) to confirm that >70% of sessions were completed, our *a priori* goal, assuming a moderate standard deviation of 15%,. For acceptability, using prior ratings for ease and utility of sessions (mean 8.1 [SD 2.3]) (Huffman et al., 2019), these studies had a minimum of 95% power to detect mean ratings of ≥7.0 for each of the ease and utility scales, the *a priori* metrics for acceptability.

### Between-study analyses

Secondary exploratory analyses examining differences in feasibility, acceptability, and impact between the BEHOLD-8 and BEHOLD-16 interventions will be conducted. Specifically, we will compare rates of session completion and ease/utility ratings between the two conditions, and we will also compare outcomes between the two PP-MI conditions at the end of the intervention and at the subsequent 8-week follow-up. Similar to between-group difference calculations in the individual studies, acceptability and feasibility data will be compared using independent samples t-tests. Changes in MVPA and additional psychological, behavioral, and medical outcomes will be compared using mixed effects regression models. All analyses will be performed using Stata 16 (StataCorp: College Station, TX) under the supervision of the study biostatistician (BH).

## Discussion

There is a substantial public health need to develop and implement scalable, easy-to-deliver behavioral interventions to promote physical activity in T2D (Carroll et al., 2015; Parikh et al., 2010; Whittemore et al., 2009). The BEHOLD pilot trials can provide important initial information about whether phone-delivered PP-MI interventions may be feasible and acceptable in inactive T2D patients. They can also provide preliminary data about whether PP-MI might provide greater benefit than relevant, health-focused, attention-matched control conditions on psychological and activity-related outcome measures.

Furthermore, conducting these two pilot trials concurrently provides an opportunity to perform exploratory secondary analyses comparing the two PP-MI interventions (BEHOLD-8 vs. BEHOLD-16). By comparing feasibility and acceptability measures, along with impact-related outcomes, we can gain an understanding about which intervention design may best fit this large and important population at elevated risk for heart disease and mortality.

Specifically, establishing a suitable dose/duration is essential at this stage, as different intervention durations can have implications for participant engagement and retention, budgeting, staffing, and effect. While there is some evidence to support greater efficacy of behavioral interventions with longer intervention duration (Lin et al., 2014), longer programs can be more costly and run the risk of lower patient engagement due to greater burden (Voils et al., 2014). Given the lack of evidence regarding these questions in behavioral change interventions aimed at increasing physical activity, this trial will allow us to rationally select an optimal intervention for a larger, NIH Stage II (Onken et al., 2014) randomized clinical trial that is powered to detect between group differences in MVPA.

These trials and analyses have important limitations. They are being conducted at a single academic medical center, with a predominantly non-Latino White population. In addition, given the nature of these trials and their sample sizes, they were not designed to make definitive assessments about intervention impact on psychological or health behavior outcomes. Finally, the between-study comparisons of interventions were not powered to detect significant differences in the two conditions, and the timing of follow-up assessments differed (e.g. follow-up 8 weeks post-intervention was at 16 and 24 weeks in BEHOLD-8 and BEHOLD-16 respectively). Despite these limitations, these studies should provide valuable information about a behavioral intervention designed to promote physical activity among inactive T2D patients, will greatly assist in selecting an intervention duration that meets the needs and preferences of persons with T2D, and will inform further trials that can more definitively answer whether such a program could be successful in this population.

## Supplementary Material

Supplemental Material

## References

[CIT0001] American Diabetes Association (2013). Standards of medical care in diabetes: 2013. *Diabetes Care*, *36*(Suppl 1), S11–S66. 23264422 10.2337/dc13-S011 PMC3537269

[CIT0002] Avery, L., Flynn, D., van Wersch, A., Sniehotta, F. F., & Trenell, M. I. (2012). Changing physical activity behavior in type 2 diabetes: A systematic review and meta-analysis of behavioral interventions. *Diabetes Care*, *35*(12), 2681–2689. 23173137 10.2337/dc11-2452 PMC3507564

[CIT0003] Bandura, A. (2004). Health promotion by social cognitive means. *Health Education & Behavior*, *31*(2), 143–164. 15090118 10.1177/1090198104263660

[CIT0004] Bandura, A., & Locke, E. A. (2003). Negative self-efficacy and goal effects revisited. *The Journal of Applied Psychology*, *88*(1), 87–99. 12675397 10.1037/0021-9010.88.1.87

[CIT0005] Baron, R. M., & Kenny, D. A. (1986). The moderator-mediator variable distinction in social psychological research: Conceptual, strategic and statistical considerations. *Journal of Personality and Social Psychology*, *51*, 1173–1182. 3806354 10.1037//0022-3514.51.6.1173

[CIT0006] Bartlett, S. J., Orbai, A. M., Duncan, T., DeLeon, E., Ruffing, V., Clegg-Smith, K., … Zhang, C. (2015). Reliability and validity of selected PROMIS measures in people with rheumatoid arthritis. *PLoS One*, *10*(9), e0138543. 26379233 10.1371/journal.pone.0138543 PMC4575023

[CIT0007] Bean, M. K., Powell, P., Quinoy, A., Ingersoll, K., Wickham, E. P., & Mazzeo, S. E. (2015). Motivational interviewing targeting diet and physical activity improves adherence to paediatric obesity treatment: Results from the MI Values randomized controlled trial. *Pediatric Obesity*, *10*(2), 118–125. 24729537 10.1111/j.2047-6310.2014.226.x PMC4197118

[CIT0008] Benjamin, E. J., Muntner, P., Alonso, A., Bittencourt, M. S., Callaway, C. W., Carson, A. P., … Delling, F. N. (2019). Heart disease and stroke statistics-2019 update: A report from the American Heart Association. *Circulation*, *139*(10), e56–e528. 30700139 10.1161/CIR.0000000000000659

[CIT0009] Bennett, J. A., Young, H. M., Nail, L. M., Winters-Stone, K., & Hanson, G. (2008). A telephone-only motivational intervention to increase physical activity in rural adults: A randomized controlled trial. *Nursing Research*, *57*(1), 24–32. 18091289 10.1097/01.NNR.0000280661.34502.c1

[CIT0010] Bjelland, I., Dahl, A. A., Haug, T. T., & Neckelmann, D. (2002). The validity of the hospital anxiety and depression scale. An updated literature review. *Journal of Psychosomatic Research*, *52*(2), 69–77. 11832252 10.1016/s0022-3999(01)00296-3

[CIT0011] Blackwell, E., de Leon, C. F., & Miller, G. E. (2006). Applying mixed regression models to the analysis of repeated-measures data in psychosomatic medicine. *Psychosomatic Medicine*, *68*(6), 870–878. 17079709 10.1097/01.psy.0000239144.91689.ca

[CIT0012] Bolier, L., Haverman, M., Westerhof, G. J., Riper, H., Smit, F., & Bohlmeijer, E. (2013). Positive psychology interventions: A meta-analysis of randomized controlled studies. *BMC Public Health*, *13*(119). https://bmcpublichealth.biomedcentral.com/articles/10.1186/1471-2458-13-119 10.1186/1471-2458-13-119 PMC3599475 23390882

[CIT0013] Booth, M. L., Ainsworth, B. E., Pratt, M., Ekelund, U., Yngve, A., Sallis, J. F., & Oja, P. (2003). International physical activity questionnaire: 12-country reliability and validity. *Medicine & Science in Sports & Exercise*, *195*(9131/03), 3508–1381. 10.1249/01.MSS.0000078924.61453.FB 12900694

[CIT0014] Boudreau, F., & Godin, G. (2014). Participation in regular leisure-time physical activity among individuals with type 2 diabetes not meeting Canadian guidelines. *International Journal of Behavioral Medicine*, *21*(6), 918–926. 24442932 10.1007/s12529-013-9380-4

[CIT0015] Bowen, D. J., Kreuter, M., Spring, B., Cofta-Woerpel, L., Linnan, L., Weiner, D., … Fernandez, M. (2009). How we design feasibility studies. *American Journal of Preventive Medicine*, *36*(5), 452–457. 19362699 10.1016/j.amepre.2009.02.002 PMC2859314

[CIT0016] Callahan, C. M., Unverzagt, F. W., Hui, S. L., Perkins, A. J., & Hendrie, H. C. (2002). Six-item screener to identify cognitive impairment among potential subjects for clinical research. *Medical Care*, *40*(9), 771–781. 12218768 10.1097/00005650-200209000-00007

[CIT0017] Carrico, A. W., Gómez, W., Siever, M. D., Discepola, M. V., Dilworth, S. E., & Moskowitz, J. T. (2015). Pilot randomized controlled trial of an integrative intervention with methamphetamine-using men who have sex with men. *Archives of Sexual Behavior*, *44*(7), 1861–1867. 26123068 10.1007/s10508-015-0505-5 PMC4560962

[CIT0018] Carroll, J., Winters, P., Fiscella, K., Williams, G., Bauch, J., Clark, L., … Bennett, N. (2015). Process evaluation of practice-based diabetes prevention programs: What are the implementation challenges? *Diabetes Educator*, *41*(3), 271–279. 10.1177/0145721715572444 25759431

[CIT0019] Celano, C. M., Albanese, A. M., Millstein, R. A., Mastromauro, C. A., Chung, W. J., Campbell, K. A., … Januzzi, J. L. (2018). Optimizing a positive psychology intervention to promote health behaviors following an acute coronary syndrome: The positive emotions after acute coronary events III (PEACE-III) randomized factorial trial. *Psychosomatic Medicine*, *80*(6), 526–534. 29624523 10.1097/PSY.0000000000000584 PMC6023730

[CIT0020] Celano, C. M., Freedman, M. E., Beale, E. E., Gomez-Bernal, F., & Huffman, J. C. (2018). A positive psychology intervention to promote health behaviors in heart Failure: A proof-of-concept trial. *The Journal of Nervous and Mental Disease*, *206*(10), 800–808. 30273277 10.1097/NMD.0000000000000883 PMC6169996

[CIT0021] Celano, C. M., Gianangelo, T. A., Millstein, R. A., Chung, W. J., Wexler, D. J., Park, E. R., & Huffman, J. C. (2019). A positive psychology-motivational interviewing intervention for patients with type 2 diabetes: Proof-of-concept trial. *The International Journal of Psychiatry in Medicine*, *54*(2), 97–114. 30114958 10.1177/0091217418791448 PMC6370502

[CIT0022] Charlson, M. E., Pompei, P., Ales, K. L., & MacKenzie, C. R. (1987). A new method of classifying prognostic comorbidity in longitudinal studies: Development and validation. *Journal of Chronic Diseases*, *40*(5), 373–383. 3558716 10.1016/0021-9681(87)90171-8

[CIT0023] Cheung, E. O., Cohn, M. A., Dunn, L. B., Melisko, M. E., Morgan, S., Penedo, F. J., … Moskowitz, J. T. (2017). A randomized pilot trial of a positive affect skill intervention (lessons in linking affect and coping) for women with metastatic breast cancer. *Psycho-oncology*, *26*(12), 2101–2108. 27862646 10.1002/pon.4312 PMC5550341

[CIT0024] Choi, L., Ward, S. C., Schnelle, J. F., & Buchowski, M. S. (2012). Assessment of wear/nonwear time classification algorithms for triaxial accelerometer. *Medicine and Science in Sports and Exercise*, *44*(10), 2009–2016. 22525772 10.1249/MSS.0b013e318258cb36 PMC3443532

[CIT0025] Christie, D., & Channon, S. (2014). The potential for motivational interviewing to improve outcomes in the management of diabetes and obesity: A clinical review. *Diabetes, Obesity and Metabolism*, *16*(5), 381–387. 10.1111/dom.12195 PMC4237607 23927612

[CIT0026] Cohn, M. A., Pietrucha, M. E., Saslow, L. R., Hult, J. R., & Moskowitz, J. T. (2014). An online positive affect skills intervention reduces depression in adults with type 2 diabetes. *The Journal of Positive Psychology*, *9*(6), 523–534. 25214877 10.1080/17439760.2014.920410 PMC4157680

[CIT0027] Colberg, S. R., Sigal, R. J., Yardley, J. E., Riddell, M. C., Dunstan, D. W., Dempsey, P. C., & Horton, E. S. (2016). Physical activity/exercise and diabetes: A position statement of the American diabetes association. *Diabetes Care*, *39*, 2065–2079. 27926890 10.2337/dc16-1728 PMC6908414

[CIT0028] Collins, L. M., Murphy, S. A., Nair, V. N., & Strecher, V. J. (2005). A strategy for optimizing and evaluating behavioral interventions. *Annals of Behavioral Medicine*, *30*(1), 65–73. 16097907 10.1207/s15324796abm3001_8

[CIT0029] Copeland, J. L., & Esliger, D. W. (2009). Accelerometer assessment of physical activity in active, healthy older adults. *Journal of Aging and Physical Activity*, *17*(1), 17–30. 19299836 10.1123/japa.17.1.17

[CIT0030] DiMatteo, M. R., & Haskard-Zolnierek, K. B. (2011). Impact of depression on treatment adherence and survival from cancer. In D. W. Kissane, M. Maj, & N. Sartorius (Eds.), *Depression and Cancer* (pp. 101–124). Hoboken, NJ: John Wiley & Sons, Ltd.

[CIT0031] Dohrn, I. M., Kwak, L., Oja, P., Sjostrom, M., & Hagstromer, M. (2018). Replacing sedentary time with physical activity: A 15-year follow-up of mortality in a national cohort. *Clinical Epidemiology*, *10*, 179–186. 29416378 10.2147/CLEP.S151613 PMC5790069

[CIT0032] Dougherty, C. M., Glenny, R. W., Kudenchuk, P. J., Malinick, T. E., & Flo, G. L. (2010). Testing an exercise intervention to improve aerobic conditioning and autonomic function after an implantable cardioverter defibrillator (ICD). *Pacing and Clinical Electrophysiology: PACE*, *33*(8), 973–980. 20230460 10.1111/j.1540-8159.2010.02728.x PMC2922694

[CIT0033] DuBois, C. M., Beach, S. R., Kashdan, T. B., Nyer, M. B., Park, E. R., Celano, C. M., & Huffman, J. C. (2012). Positive psychological attributes and cardiac outcomes: Associations, mechanisms, and interventions. *Psychosomatics*, *53*(4), 303–318. 22748749 10.1016/j.psym.2012.04.004

[CIT0034] DuBois, C. M., Millstein, R. A., Celano, C. M., Wexler, D. J., & Huffman, J. C. (2016). Feasibility and acceptability of a positive psychological intervention for patients With type 2 diabetes. *The Primary Care Companion for CNS Disorders*, *18*(3). 10.4088/PCC.15m01902 PMC5035810 27733954

[CIT0035] Duque, L., Brown, L., Celano, C. M., Healy, B., & Huffman, J. C. (2019). Is it better to cultivate positive affect or optimism? Predicting improvements in medical adherence following a positive psychology intervention in patients with acute coronary syndrome. *General Hospital Psychiatry*, *61*, 125–129. 31280918 10.1016/j.genhosppsych.2019.06.001 PMC6861647

[CIT0036] Dutton, G. R., Tan, F., Provost, B. C., Sorenson, J. L., Allen, B., & Smith, D. (2009). Relationship between self-efficacy and physical activity among patients with type 2 diabetes. *Journal of Behavioral Medicine*, *32*(3), 270–277. 19156510 10.1007/s10865-009-9200-0

[CIT0037] Emmons, R. A., & McCullough, M. E. (2003). Counting blessings versus burdens: An experimental investigation of gratitude and subjective well-being in daily life. *Journal of Personality and Social Psychology*, *84*(2), 377–389. 12585811 10.1037//0022-3514.84.2.377

[CIT0038] Fredrickson, B. L. (2001). The role of positive emotions in positive psychology: The broaden-and-build theory of positive emotions. *American Psychologist*, *56*(3), 218–226. 10.1037//0003-066x.56.3.218 PMC3122271 11315248

[CIT0039] Fredrickson, B. L., Cohn, M. A., Coffey, K. A., Pek, J., & Finkel, S. M. (2008). Open hearts build lives: Positive emotions, induced through loving-kindness meditation, build consequential personal resources. *Journal of Personality and Social Psychology*, *95*(5), 1045–1062. 18954193 10.1037/a0013262 PMC3156028

[CIT0040] Gebel, K., Ding, D., Chey, T., Stamatakis, E., Brown, W. J., & Bauman, A. E. (2015). Effect of moderate to vigorous physical activity on all-cause mortality in middle-aged and older Australians. *JAMA Internal Medicine*, *175*(6), 970–977. 25844882 10.1001/jamainternmed.2015.0541

[CIT0041] Giltay, E. J., Geleijnse, J. M., Zitman, F. G., Buijsse, B., & Kromhout, D. (2007). Lifestyle and dietary correlates of dispositional optimism in men: The Zutphen elderly study. *Journal of Psychosomatic Research*, *63*(5), 483–490. 17980220 10.1016/j.jpsychores.2007.07.014

[CIT0042] Goldstein, M. G., Whitlock, E. P., & DePue, J. (2004). Multiple behavioral risk factor interventions in primary care: Summary of research evidence. *American Journal of Preventive Medicine*, *27*(2 Suppl), 61–79. 15275675 10.1016/j.amepre.2004.04.023

[CIT0043] Goossens, M. E., Vlaeyen, J. W., Hidding, A., Kole-Snijders, A., & Evers, S. M. (2005). Treatment expectancy affects the outcome of cognitive-behavioral interventions in chronic pain. *The Clinical Journal of Pain*, *21*(1), 18–26. 15599128 10.1097/00002508-200501000-00003

[CIT0044] Gorman, E., Hanson, H. M., Yang, P. H., Khan, K. M., Liu-Ambrose, T., & Ashe, M. C. (2014). Accelerometry analysis of physical activity and sedentary behavior in older adults: A systematic review and data analysis. *European Review of Aging and Physical Activity*, *11*, 35–49. 24765212 10.1007/s11556-013-0132-x PMC3990855

[CIT0045] Gourlan, M., Sarrazin, P., & Trouilloud, D. (2013). Motivational interviewing as a way to promote physical activity in obese adolescents: A randomised-controlled trial using self-determination theory as an explanatory framework. *Psychology & Health*, *28*(11), 1265–1286. 23756082 10.1080/08870446.2013.800518

[CIT0046] Han, B., Enas, N. H., & McEntegart, D. (2009). Randomization by minimization for unbalanced treatment allocation. *Statistics in Medicine*, *28*(27), 3329–3346. 19739238 10.1002/sim.3710

[CIT0047] He, F., Cao, R., Feng, Z., Guan, H., & Peng, J. (2013). The impacts of dispositional optimism and psychological resilience on the subjective well-being of burn patients: A structural equation modelling analysis. *PLoS One*, *8*(12), e82939. 24358241 10.1371/journal.pone.0082939 PMC3866201

[CIT0048] Hoffmann, T. C., Glasziou, P. P., Boutron, I., Milne, R., Perera, R., Moher, D., … Michie, S. (2014). Better reporting of interventions: Template for intervention description and replication (TIDieR) checklist and guide. *BMJ*, *348*, g1687. 24609605 10.1136/bmj.g1687

[CIT0049] Huffman, J. C., Albanese, A. M., Campbell, K. A., Celano, C. M., Millstein, R. A., Mastromauro, C. A., … Park, E. R. (2017). The positive emotions after acute coronary events behavioral health intervention: Design, rationale, and preliminary feasibility of a factorial design study. *Clinical Trials*, *14*(2), 128–139. 28079394 10.1177/1740774516673365 PMC5376225

[CIT0050] Huffman, J. C., Beale, E. E., Celano, C. M., Beach, S. R., Belcher, A. M., Moore, S. V., … Januzzi, J. L. (2016). Effects of optimism and gratitude on physical activity, biomarkers, and readmissions after an acute coronary syndrome: The gratitude research in acute coronary events study. *Circulation: Cardiovascular Quality and Outcomes*, *9*(1), 55–63. 26646818 10.1161/CIRCOUTCOMES.115.002184 PMC4720551

[CIT0051] Huffman, J. C., DuBois, C. M., Millstein, R. A., Celano, C. M., & Wexler, D. (2015). Positive psychological interventions for patients with type 2 diabetes: Rationale, theoretical model, and intervention development. *Journal of Diabetes Research*, *2015*. 10.1155/2015/428349 PMC4442018 26064980

[CIT0052] Huffman, J. C., Feig, E. H., Millstein, R. A., Freedman, M., Healy, B. C., Chung, W. J., … Celano, C. M. (2019). Usefulness of a positive psychology-motivational interviewing intervention to promote positive affect and physical activity after an Acute coronary Syndrome. *The American Journal of Cardiology*, *123*(12), 1906–1914. 30979409 10.1016/j.amjcard.2019.03.023 PMC6529259

[CIT0053] Huffman, J. C., Mastromauro, C. A., Boehm, J. K., Seabrook, R., Fricchione, G. L., Denninger, J. W., & Lyubomirsky, S. (2011). Development of a positive psychology intervention for patients with acute cardiovascular disease. *Heart International*, *6*(2), e14. 23825741 10.4081/hi.2011.e14 PMC3699107

[CIT0054] Huo, X., Gao, L., Guo, L., Xu, W., Wang, W., Zhi, X., … Ji, L. (2016). Risk of non-fatal cardiovascular diseases in early-onset versus late-onset type 2 diabetes in China: A cross-sectional study. *The Lancet Diabetes & Endocrinology*, *4*(2), 115–124. 26704379 10.1016/S2213-8587(15)00508-2

[CIT0055] Ingersoll, K. S., Banton, T., Gorlin, E., Vajda, K., Singh, H., Peterson, N., … Cox, D. J. (2015). Motivational interviewing support for a behavioral health internet intervention for drivers with type 1 diabetes. *Internet Interventions*, *2*(2), 103–109. 25774342 10.1016/j.invent.2015.02.001 PMC4356504

[CIT0056] !!! INVALID CITATION !!! (3, 4).

[CIT0057] Ismail, K., Maissi, E., Thomas, S., Chalder, T., Schmidt, U., Bartlett, J., … Treasure, J. (2010). A randomised controlled trial of cognitive behaviour therapy and motivational interviewing for people with type 1 diabetes mellitus with persistent sub-optimal glycaemic control. *Health Technology Assessment*, *14*(22), 1–101. 10.3310/hta14220 20483060

[CIT0058] Joseph, C. L., Havstad, S. L., Johnson, D., Saltzgaber, J., Peterson, E. L., Resnicow, K., … Strecher, V. J. (2010). Factors associated with nonresponse to a computer-tailored asthma management program for urban adolescents with asthma. *The Journal of Asthma: Official Journal of the Association for the Care of Asthma*, *47*(6), 667–673. 20642376 10.3109/02770900903518827 PMC4017358

[CIT0059] Kealey, K. A., Ludman, E. J., Marek, P. M., Mann, S. L., Bricker, J. B., & Peterson, A. V. (2009). Design and implementation of an effective telephone counseling intervention for adolescent smoking cessation. *JNCI: Journal of the National Cancer Institute*, *101*(20), 1393–1405. 19822837 10.1093/jnci/djp318 PMC2765262

[CIT0060] King, L. A. (2001). The health benefits of writing about life goals. *Personality and Social Psychology Bulletin*, *27*, 10–17.

[CIT0061] Kubota, Y., Evenson, K. R., Maclehose, R. F., Roetker, N. S., Joshu, C. E., & Folsom, A. R. (2017). Physical activity and lifetime risk of cardiovascular disease and cancer. *Medicine & Science in Sports & Exercise*, *49*(8), 1599–1605. 28350711 10.1249/MSS.0000000000001274 PMC5511058

[CIT0062] Lee, P. H., Macfarlane, D. J., Lam, T., & Stewart, S. M. (2011). Validity of the international physical activity questionnaire short form (IPAQ-SF): A systematic review. *International Journal of Behavioral Nutrition and Physical Activity*, *8*(1), 1. 10.1186/1479-5868-8-115 PMC3214824 22018588

[CIT0063] Lee, V., Robin Cohen, S., Edgar, L., Laizner, A. M., & Gagnon, A. J. (2006). Meaning-making intervention during breast or colorectal cancer treatment improves self-esteem, optimism, and self-efficacy. *Social Science & Medicine*, *62*(12), 3133–3145. 16413644 10.1016/j.socscimed.2005.11.041

[CIT0064] Lilienthal, K. R., Pignol, A. E., Holm, J. E., & Vogeltanz-Holm, N. (2014). Telephone-based motivational interviewing to promote physical activity and stage of change progression in older adults. *Journal of Aging and Physical Activity*, *22*(4), 527–535. 24226309 10.1123/japa.2013-0056

[CIT0065] Lin, J. S., O'Connor, E., Evans, C. V., Senger, C. A., Rowland, M. G., & Groom, H. C. (2014). Behavioral counseling to promote a healthy lifestyle in persons with cardiovascular risk factors: A systematic review for the U.S. Preventive Services Task Force. *Annals of Internal Medicine*, *161*(8), 568–578. 25155549 10.7326/M14-0130

[CIT0066] Littlecott, H. J., Moore, G. F., Moore, L., & Murphy, S. (2014). Psychosocial mediators of change in physical activity in the Welsh national exercise referral scheme: Secondary analysis of a randomised controlled trial. *International Journal of Behavioral Nutrition and Physical Activity*, *11*, 109. 10.1186/s12966-014-0109-9 PMC4173052 25209188

[CIT0067] Loprinzi, P. D. (2017). Light-intensity physical activity and all-cause mortality. *American Journal of Health Promotion*, *31*(4), 340–342. 26730555 10.4278/ajhp.150515-ARB-882

[CIT0068] Lynch, B. M., Boyle, T., Winkler, E., Occleston, J., Courneya, K. S., & Vallance, J. K. (2016). Patterns and correlates of accelerometer-assessed physical activity and sedentary time among colon cancer survivors. *Cancer Causes & Control: CCC*, *27*(1), 59–68. 26518196 10.1007/s10552-015-0683-4

[CIT0069] Lyubomirsky, S., & Layous, K. (2013). How do simple positive activities increase well-being? *Current Directions in Psychological Science*, *22*(1), 57–62.

[CIT0070] Madva, E. N., Gomez-Bernal, F., Millstein, R. A., Celano, C. M., Park, E. R., Mastromauro, C. A., … Huffman, J. C. (2018). Magnitude and sources of distress in mid-life adults with chronic medical illness: An exploratory mixed-methods analysis. *Psychology, Health & Medicine*, *23*(5), 555–566. 10.1080/13548506.2017.1384554 PMC6186490 28984158

[CIT0071] Majer, J. M., Jason, L. A., & Olson, B. D. (2004). Optimism, abstinence self-efficacy, and self-mastery: A comparative analysis of cognitive resources. *Assessment*, *11*(1), 57–63. 14994954 10.1177/1073191103257139

[CIT0072] Martins, R. K., & McNeil, D. W. (2009). Review of motivational interviewing in promoting health behaviors. *Clinical Psychology Review*, *29*(4), 283–293. 19328605 10.1016/j.cpr.2009.02.001

[CIT0073] Matthews, L., Kirk, A., Macmillan, F., & Mutrie, N. (2014). Can physical activity interventions for adults with type 2 diabetes be translated into practice settings? A systematic review using the RE-AIM framework. *Translational Behavioral Medicine*, *4*(1), 60–78. 24653777 10.1007/s13142-013-0235-y PMC3958594

[CIT0074] Meevissen, Y. M., Peters, M. L., & Alberts, H. J. (2011). Become more optimistic by imagining a best possible self: Effects of a two week intervention. *Journal of Behavior Therapy and Experimental Psychiatry*, *42*(3), 371–378. 21450262 10.1016/j.jbtep.2011.02.012

[CIT0075] Miller, W. R., & Rollnick, S. (2012a). *Motivational interviewing: Helping people change*. New York: Guilford Press.

[CIT0076] Miller, W. R., & Rollnick, S. (2012b). *Motivational interviewing: Preparing people for change* (3rd ed.). New York, NY: Guilford Press.

[CIT0077] Moreau, M., Gagnon, M. P., & Boudreau, F. (2015). Development of a fully automated, web-based, tailored intervention promoting regular physical activity among insufficiently active adults with type 2 diabetes: Integrating the I-change model, self-determination theory, and motivational interviewing components. *Journal of Medical Internet Research*, *4*(1), e25. 10.2196/resprot.4099 PMC4376153 25691346

[CIT0078] Morrato, E. H., Hill, J. O., Wyatt, H. R., Ghushchyan, V., & Sullivan, P. W. (2007). Physical activity in U.S. adults with diabetes and at risk for developing diabetes, 2003. *Diabetes Care*, *30*(2), 203–209. 17259482 10.2337/dc06-1128

[CIT0079] Moskowitz, J. T., Epel, E. S., & Acree, M. (2008). Positive affect uniquely predicts lower risk of mortality in people with diabetes. *Health Psychology*, *27*(1 Suppl), S73–S82. 18248108 10.1037/0278-6133.27.1.S73

[CIT0080] Moskowitz, J. T., Hult, J. R., Duncan, L. G., Cohn, M. A., Maurer, S., Bussolari, C., & Acree, M. (2012). A positive affect intervention for people experiencing health-related stress: Development and non-randomized pilot test. *Journal of Health Psychology*, *17*(5), 676–692. 22021272 10.1177/1359105311425275 PMC3498769

[CIT0081] Moyers, T. B., Rowell, L. N., Manuel, J. K., Ernst, D., & Houck, J. M. (2016). The Motivational Interviewing Treatment Integrity Code (MITI 4): Rationale, preliminary reliability and validity. *Journal of Substance Abuse Treatment*, *65*, 36–42. 26874558 10.1016/j.jsat.2016.01.001 PMC5539964

[CIT0082] Nikrahan, G. R., Eshaghi, L., Massey, C. N., Hemmat, A., Amonoo, H. L., Healy, B., & Huffman, J. C. (2019). Randomized controlled trial of a well-being intervention in cardiac patients. *General Hospital Psychiatry*, *61*, 116–124. 31285062 10.1016/j.genhosppsych.2019.06.005

[CIT0083] Ogedegbe, G. O., Boutin-Foster, C., Wells, M. T., Allegrante, J. P., Isen, A. M., Jobe, J. B., & Charlson, M. E. (2012). A randomized controlled trial of positive-affect intervention and medication adherence in hypertensive African Americans. *Archives of Internal Medicine*, *172*(4), 322–326. 22269592 10.1001/archinternmed.2011.1307 PMC4669680

[CIT0084] Onken, L. S., Carroll, K. M., Shoham, V., Cuthbert, B. N., & Riddle, M. (2014). Reenvisioning clinical science: Unifying the discipline to improve the public health. *Clinical Psychological Science*, *2*(1), 22–34. 25821658 10.1177/2167702613497932 PMC4374633

[CIT0085] Parikh, P., Simon, E. P., Fei, K., Looker, H., Goytia, C., & Horowitz, C. R. (2010). Results of a pilot diabetes prevention intervention in East Harlem, New York City: Project HEED. *American Journal of Public Health*, *100*(Suppl 1), S232–S239. 20147680 10.2105/AJPH.2009.170910 PMC2837455

[CIT0086] Peterson, J. C., Charlson, M. E., Hoffman, Z., Wells, M. T., Wong, S. C., Hollenberg, J. P., … Allegrante, J. P. (2012). A randomized controlled trial of positive-affect induction to promote physical activity after percutaneous coronary intervention. *Archives of Internal Medicine*, *172*(4), 329–336. 22269589 10.1001/archinternmed.2011.1311 PMC3717982

[CIT0087] Plotnikoff, R. C., Trinh, L., Courneya, K. S., Karunamuni, N., & Sigal, R. J. (2011). Predictors of physical activity in adults with type 2 diabetes. *American Journal of Health Behavior*, *35*(3), 359–370. 21683024 10.5993/ajhb.35.3.9

[CIT0088] Prince, S. A., Blanchard, C. M., Grace, S. L., & Reid, R. D. (2016). Objectively-measured sedentary time and its association with markers of cardiometabolic health and fitness among cardiac rehabilitation graduates. *European Journal of Preventive Cardiology*, *23*, 818–825. 26607698 10.1177/2047487315617101

[CIT0089] Resnick, B., & Jenkins, L. S. (2000). Testing the reliability and validity of the self-efficacy for exercise scale. *Nursing Research*, *49*(3), 154–159. 10882320 10.1097/00006199-200005000-00007

[CIT0090] Robb, S. L., Burns, D. S., Docherty, S. L., & Haase, J. E. (2011). Ensuring treatment fidelity in a multi-site behavioral intervention study: Implementing NIH behavior change Consortium recommendations in the SMART trial. *Psycho-oncology*, *20*(11), 1193–1201. 22012943 10.1002/pon.1845 PMC3198011

[CIT0091] Safren, S. A., Gonzalez, J. S., Wexler, D. J., Psaros, C., Delahanty, L. M., Blashill, A. J., … Cagliero, E. (2014). A randomized controlled trial of cognitive behavioral therapy for adherence and depression (CBT-AD) in patients with uncontrolled type 2 diabetes. *Diabetes Care*, *37*(3), 625–633. 24170758 10.2337/dc13-0816 PMC3931377

[CIT0092] Saghaei, M. (2011). An overview of randomization and minimization programs for randomized clinical trials. *Journal of Medical Signals and Sensors*, *1*(1), 55–61. 22606659 PMC3317766

[CIT0093] Saint-Maurice, P. F., Troiano, R. P., Matthews, C. E., & Kraus, W. E. (2018). Moderate-to-vigorous physical activity and All-cause mortality: Do bouts matter? *Journal of the American Heart Association*, *7*(6). 10.1161/JAHA.117.007678 PMC5907548 29567764

[CIT0094] Scheier, M. F., Carver, C. S., & Bridges, M. W. (1994). Distinguishing optimism from neuroticism (and trait anxiety, self-mastery, and self-esteem): A reevaluation of the life orientation test. *Journal of Personality and Social Psychology*, *67*(6), 1063–1078. 7815302 10.1037//0022-3514.67.6.1063

[CIT0095] Scheier, M. F., Helgeson, V. S., Schulz, R., Colvin, S., Berga, S. L., Knapp, J., & Gerszten, K. (2007). Moderators of interventions designed to enhance physical and psychological functioning among younger women with early-stage breast cancer. *Journal of Clinical Oncology*, *25*(36), 5710–5714. 17998547 10.1200/JCO.2007.11.7093

[CIT0096] Scheier, M. F., Matthews, K. A., Owens, J. F., Magovern, G. J., Lefebvre, R. C., Abbott, R. A., & Carver, C. S. (1989). Dispositional optimism and recovery from coronary artery bypass surgery. *Journal of Personality and Social Psychology*, *57*(6), 1024–1040. 2614656 10.1037//0022-3514.57.6.1024

[CIT0097] Schmiege, S. J., Feldstein Ewing, S. W., Hendershot, C. S., & Bryan, A. D. (2011). Positive outlook as a moderator of the effectiveness of an HIV/STI intervention with adolescents in detention. *Health Education Research*, *26*(3), 432–442. 20926554 10.1093/her/cyq060 PMC3099182

[CIT0098] Schneider, K. L., Pagoto, S. L., Handschin, B., Panza, E., Bakke, S., Liu, Q., … Ma, Y. (2011). Design and methods for a pilot randomized clinical trial involving exercise and behavioral activation to treat comorbid type 2 diabetes and major depressive disorder. *Mental Health and Physical Activity*, *4*(1), 13–21. 21765864 10.1016/j.mhpa.2011.04.001 PMC3134367

[CIT0099] Schueller, S. M., & Parks, A. C. (2012). Disseminating self-help: Positive psychology exercises in an online trial. *Journal of Medical Internet Research*, *14*(3), e63. 22732765 10.2196/jmir.1850 PMC3414858

[CIT0100] Scott, N. W., McPherson, G. C., Ramsay, C. R., & Campbell, M. K. (2002). The method of minimization for allocation to clinical trials: A review. *Controlled Clinical Trials*, *23*(6), 662–674. 12505244 10.1016/s0197-2456(02)00242-8

[CIT0101] Smeets, E., Neff, K., Alberts, H., & Peters, M. (2014). Meeting suffering with kindness: Effects of a brief self-compassion intervention for female college students. *Journal of Clinical Psychology*, *70*(9), 794–807. 24691680 10.1002/jclp.22076

[CIT0102] Smith, B. W., Dalen, J., Wiggins, K., Tooley, E., Christopher, P., & Bernard, J. (2008). The brief resilience scale: Assessing the ability to bounce back. *International Journal of Behavioral Medicine*, *15*(3), 194–200. 18696313 10.1080/10705500802222972

[CIT0103] Smith, D. E., Kratt, P. P., & Mason, D. A. (1997). Motivational interviewing to improve adherence to a behavioral weight-control program for older obese women with NIDDM: A pilot study. *Diabetes Care*, *20*(1), 52–54. 9028693 10.2337/diacare.20.1.52

[CIT0104] Soderlund, P. D. (2018). Effectiveness of motivational interviewing for improving physical activity self-management for adults with type 2 diabetes: A review. *Chronic Illness*, *14*(1), 54–68. 29226694 10.1177/1742395317699449

[CIT0105] Steptoe, A., Wright, C., Kunz-Ebrecht, S. R., & Iliffe, S. (2006). Dispositional optimism and health behaviour in community-dwelling older people: Associations with healthy ageing. *British Journal of Health Psychology*, *11*(Pt 1), 71–84. 16480556 10.1348/135910705X42850

[CIT0106] Sweet, S. N., Fortier, M. S., Guerin, E., Tulloch, H., Sigal, R. J., Kenny, G. P., & Reid, R. D. (2009). Understanding physical activity in adults with type 2 diabetes after completing an exercise intervention trial: A mediation model of self-efficacy and autonomous motivation. *Psychology, Health & Medicine*, *14*(4), 419–429. 10.1080/13548500903111806 19697252

[CIT0107] Taves, D. R. (1974). Minimization: A new method of assigning patients to treatment and control groups. *Clinical Pharmacology & Therapeutics*, *15*(5), 443–453. 4597226 10.1002/cpt1974155443

[CIT0108] Voils, C. I., King, H. A., Maciejewski, M. L., Allen, K. D., Yancy Jr, W. S., & Shaffer, J. A. (2014). Approaches for informing optimal dose of behavioral interventions. *Annals of Behavioral Medicine*, *48*(3), 392–401. 24722964 10.1007/s12160-014-9618-7 PMC4414086

[CIT0109] Watson, D., Clark, L. A., & Tellegen, A. (1988). Development and validation of brief measures of positive and negative affect: The PANAS scales. *Journal of Personality and Social Psychology*, *54*(6), 1063–1070. 3397865 10.1037//0022-3514.54.6.1063

[CIT0110] Whittemore, R., Melkus, G., Wagner, J., Dziura, J., Northrup, V., & Grey, M. (2009). Translating the diabetes prevention program to primary care: A pilot study. *Nursing Research*, *58*(1), 2–12. 19092550 10.1097/NNR.0b013e31818fcef3 PMC2689783

[CIT0111] Zelen, M. (1979). A new design for randomized clinical trials. *New England Journal of Medicine*, *300*(22), 1242–1245. 10.1056/NEJM197905313002203 431682

