# Supplementary material for: Exploring the feasibility and impact of positive psychology-motivational interviewing interventions to promote positive affect and physical activity in type 2 diabetes: design and methods from the BEHOLD-8 and BEHOLD-16 clinical trials
Source: Health Psychol Behav Med. 2020 Sep 14;8(1):398–422. doi: 10.1080/21642850.2020.1815538 (PMC7986224; doi:10.1080/21642850.2020.1815538)
Supplement: Supplemental Material [file RHPB_A_1815538_SM1101.docx]

**Supplemental table 1. The TIDieR (Template for Intervention Description and Replication) Checklist:**

Information to include when describing an intervention and the location of the information

| **Item number** | **Item** | **Where located** | | | | | |
| --- | --- | --- | --- | --- | --- | --- | --- |
|  |  | Primary paper (page or appendix number).  (Other ^†^ (details)) | | | | | |
|  |  |  | **BEHOLD-8 PPMI** | **BEHOLD-8 MI** | **BEHOLD-16 PP-MI** | **BEHOLD-16 HE** |  |
|  | **BRIEF NAME** | |  |  |  |  |  |
| **1.** | Provide the name or a phrase that describes the intervention. | | p 6 | p 6 | p 6 | p 6 |  |
|  | **WHY** |  |  |  |  |  |  |
| **2.** | Describe any rationale, theory, or goal of the elements essential to the intervention. | | p 9-11, 39 | p 9-11, 39 | p 9-11, 39 | p 9-11, 39 |  |
|  | **WHAT** |  |  |  |  |  |  |
| **3.** | Materials: Describe any physical or informational materials used in the intervention, including those provided to participants or used in intervention delivery or in training of intervention providers. Provide information on where the materials can be accessed (e.g. online appendix, URL). | | P 12 (^†^manual available from authors) | P 12 (^†^manual available from authors) | P 12 (^†^manual available from authors) | P 12 (^†^manual available from authors) |  |
| **4.** | Procedures: Describe each of the procedures, activities, and/or processes used in the intervention, including any enabling or support activities. | | P 12-14, 34 | P 14, 35 | P 15, 36 | P 15-16, 38 |  |
|  | **WHO PROVIDED** |  |  |  |  |  |  |
| **5.** | For each category of intervention provider (e.g. psychologist, nursing assistant), describe their expertise, background and any specific training given. | | P 16-17 | P 16-17 | P 16-17 | P 16-17 |  |
|  | **HOW** |  |  |  |  |  |  |
| **6.** | Describe the modes of delivery (e.g. face-to-face or by some other mechanism, such as internet or telephone) of the intervention and whether it was provided individually or in a group. | | 12 | 12, 14 | 12, 15 | 12, 15 |  |
|  | **WHERE** |  |  |  |  |  |  |
| **7.** | Describe the type(s) of location(s) where the intervention occurred, including any necessary infrastructure or relevant features. | | 12 | 12, 14 | 12, 15 | 12, 15 |  |
|  | **WHEN and HOW MUCH** |  |  |  |  |  |  |
| **8.** | Describe the number of times the intervention was delivered and over what period of time including the number of sessions, their schedule, and their duration, intensity or dose. | | P 12-14, 34, 40 | P 14, 35, 40 | P 15, 36, 40 | P 15-16, 38, 40 |  |
|  | **TAILORING** |  |  |  |  |  |  |
| **9.** | If the intervention was planned to be personalised, titrated or adapted, then describe what, why, when, and how. | | P 13-14, 34 | P 14, 35 | P 15, 36 | P 38 |  |
|  | **MODIFICATIONS** |  |  |  |  |  |  |
| **10.^ǂ^** | If the intervention was modified during the course of the study, describe the changes (what, why, when, and how). | | N/A | N/A | N/A | N/A |  |
|  | **HOW WELL** |  |  |  |  |  |  |
| **11.** | Planned: If intervention adherence or fidelity was assessed, describe how and by whom, and if any strategies were used to maintain or improve fidelity, describe them. | | P 16-17 | P 16-17 | P 16-17 | P 16-17 |  |
| **12.^ǂ^** | Actual: If intervention adherence or fidelity was assessed, describe the extent to which the intervention was delivered as planned. | | ǂ | ǂ | ǂ | ǂ |  |

PPMI means Positive psychology and Motivational Interviewing, MI: motivational interviewing, HE: health education

N/A: item is not applicable for the intervention being described.

† If the information is not provided in the primary paper, give details of where this information is available. This may include locations such as a published protocol or other published papers (provide citation details) or a website (provide the URL).

ǂ If completing the TIDieR checklist for a protocol, these items are not relevant to the protocol and cannot be described until the study is complete.
